# Hair Dyes

**Published:** 1871-03

**Authors:** 


### HAIR DYES.

So many of them are pernicious, causing convulsions and other maladies, from the poisonous quality of the lead contained in them, it would be well for those who are ashamed of gray hairs to first try, patiently, distilled water and sulphur, which, in some cases, has turned gray hair to its original color of black or brown in a few days, — so said. A substitute for distilled water is common water well boiled, or freshly fallen rain-water. The amount of powdered or "flowers of sulphur" put in the water is not material, for the water will take up a certain quantity of the sulphur, and no more, as it does of sugar in our tea-cups, the remainder falling to the bottom. This is what is meant by a "saturated solution."

